# Impact of lower challenge doses of enterotoxigenic *Escherichia coli* on clinical outcome, intestinal colonization and immune responses in adult volunteers

**DOI:** 10.1371/journal.pntd.0006442

**Published:** 2018-04-27

**Authors:** Subhra Chakraborty, Clayton Harro, Barbara DeNearing, Jessica Brubaker, Sean Connor, Nicole Maier, Len Dally, Jorge Flores, A. Louis Bourgeois, Richard Walker, David A. Sack

**Affiliations:** 1 Department of International Health, Johns Hopkins Bloomberg School of Public Health, Baltimore, Maryland, United States of America; 2 PATH, Washington, DC, United States of America; 3 The EMMES Corporation, Rockville, Maryland, United States of America; Oxford University Clinical Research Unit, VIET NAM

## Abstract

A reliable and effective human challenge model is needed to help down-select the most promising ETEC vaccines currently under development. Such a model would need to reliably induce diarrhea in a high proportion of volunteers using the lowest possible inoculum to maximize safety and sensitivity. Previously we validated a challenge model that utilized a dose of 2x10^7^ CFU of ETEC strain H10407 (LT^+^, ST^+^, CFA/I^+^ and O78^+^) to induce attack rates for moderate to severe diarrhea (MSD) of ~60–70%. Here we detail efforts to further refine the model in an attempt to determine if a lower challenge dose of H10407 can be used. Thirty subjects were randomized 1:1 to receive an oral administration of H10407 at doses of 10^6^ or 10^5^ CFU in bicarbonate buffer. After challenge, subjects were monitored for signs and symptoms of enteric illness and stool samples were collected to detect shedding of the challenge strain. Systemic and mucosal immune responses were measured using serum, antibody in lymphocyte supernatant and fecal samples. The attack rate was 13.3% (2/15) and 26.7% (4/15) for MSD in the 10^5^ and 10^6^ groups, respectively. Four MSD cases met criteria for early antibiotic treatment. All subjects but one shed the challenge strain in fecal samples. The frequency and magnitude of anti-LT toxin, CFA/I and LPS O78 immune responses were antigen, dose, severity of diarrhea and shedding levels dependent. Notably, although of lower magnitude, there were considerable immune responses in the subjects with no diarrhea. This may indicate that immune responses to asymptomatic infections of ETEC in children in the endemic countries may contribute to protection. Based on this and our prior studies, we conclude that a dose of 2x10^7^ H10407 remains the lowest practical dose for use in future volunteer studies evaluating candidate vaccines and other preventive or therapeutic ETEC interventions.

**Trial registration:** ClinicalTrials.gov NCT00844493.

## Author summary

Human challenge models can provide a platform for the initial evaluation of vaccine efficacy to down-select vaccine candidates before more expensive phase III trials are conducted. An ideal challenge model would need to reproducibly induce diarrhea in a high proportion of volunteers using the lowest possible inoculum to better maximize the sensitivity of the model. In this study, we attempted to refine the ETEC H10407 challenge model by characterizing the clinical illness induced by lower ETEC challenge doses. We also performed a comprehensive dose and clinical diarrhea severity dependent assessment of the immune responses. We found that some volunteers do become ill when challenged with these low doses, but the rates of moderate to severe diarrhea observed with these doses were too low to be useful for such volunteer studies. Therefore, we conclude a dose of 2x10^7^ H10407 remains the lowest practical dose when evaluating candidate vaccines or other preventive or therapeutic ETEC interventions. Diarrheal illness, when it occurred, was associated with higher fecal shedding levels of H10407. The magnitudes of immune responses were dependent on the dose, antigen and severity of diarrhea. This new knowledge may contribute to a better overall understanding of the immune responses to ETEC and its relation to diarrhea severity which will help facilitate and guide ETEC vaccine development strategies.

## Introduction

Enterotoxigenic *Escherichia coli* (ETEC) remain among the most common bacterial causes of diarrhea-associated morbidity and mortality [1–4]. In Africa and Southeast Asia, ETEC is also found to be an important cause of morbidity and mortality in age-groups older than 5 years of age [5]. Studies have shown that children infected with ETEC are at higher risk of becoming stunted [3, 6, 7].

As currently understood, the key virulence factors for ETEC are colonization factor antigens (CFs) and enterotoxins. CFs mediate bacterial attachment to host small intestinal epithelial cells and subsequent colonization, whereas enterotoxins including heat-labile (LT) and heat-stable (ST) toxins, occurring as either LT or ST alone or both LT/ST, disrupt fluid homeostasis in host epithelial cells, which leads to fluid and electrolyte hypersecretion and diarrhea [2, 8].

ETEC vaccine development has been a long standing WHO priority [9]. However, there are no licensed vaccines for ETEC are currently available but several approaches to develop an effective vaccine are underway. A significant challenge to successful vaccine development is our poor understanding of the immune responses that correlate best with protection against ETEC illness. Although subclinical ETEC infections are very common in endemic countries, there is a gap of knowledge regarding the breadth and magnitude of immune responses to ETEC in asymptomatic cases which may prove helpful in guiding ETEC vaccine development strategies going forward.

Historically, the ETEC human challenge model has been considered an important tool in facilitating decisions regarding the most promising ETEC vaccines. The human challenge models can provide a platform for the evaluation and screening of vaccine efficacy before more expensive and more long-term field trials are conducted in at-risk age-groups in low and middle income countries and in travelers. To maximize its utility in this regard, it is essential that the model be optimized to ensure its reliability as a decision making tool. An ideal challenge model would reproducibly induce diarrhea in a high proportion of volunteers (>50%) using the lowest possible inoculum to better maximize the sensitivity of the model, particularly in the evaluation of preventive or therapeutic interventions. If the attack rate is too low, a much larger sample size will be needed. However, an excessively high dose might achieve high attack rates but increase the risk that these high challenge doses could overwhelm any vaccine-induced protective immunity and lead to the down selection of vaccines that might have potential as public health tools [9, 10,11]. Lower ETEC doses (<10^8^) yielded inconsistent attack rates [10, 12, 13]; however, lower challenge doses have been used effectively for other well-established enteric disease challenge models, like cholera, *Shigella* and Campylobacter [14, 15, 16].

In previous studies we validated a challenge model which utilizes an ETEC dose of 2x10^7^ CFU of virulent ETEC strain H10407 with an overnight fast prior to challenge that resulted in MSD attack rates of 60–70% [8, 17]. Our earlier studies also demonstrated that persons who had previously developed diarrhea following challenge were protected from illness when re-challenged with the homologous strain [8, 17].

In this study, we attempted to further refine the challenge model by characterizing the clinical illness induced by lower ETEC challenge doses of 10^6^ and 10^5^ CFU. We also performed a comprehensive assessment of the serum and mucosal antibody responses induced by these lower challenge doses of ETEC as well as the shedding levels of the challenge organism in fecal samples from challenged subjects. In addition, to understand the kinetics and magnitudes of immune responses in the subjects with asymptomatic infection we compared antigen specific responses in subjects with MSD versus those who did not develop diarrheal illness post-challenge.

## Materials and methods

### Regulatory approval

The protocol was conducted under BB-IND 12,243 at the Center for Immunization Research (CIR), Johns Hopkins Bloomberg School of Public Health (JHBSPH). Approval to conduct the study was provided by the Western Institutional Review Board (Olympia, WA) for JHBSPH and PATH and by the Institutional Biosafety Committee of the Johns Hopkins Institutions. The study was registered in clinicalTrials.gov under NCT00844493, (for detailed protocol https://clinicaltrials.gov/ct2/show/NCT00844493).

### Study subjects

Healthy, 18- to 45-year-old, male or female subjects were recruited for the study. The study was explained to subjects in detail and signed, witnessed consent was obtained. The pre-challenge health status of subjects was assessed by written and oral medical history, physical examination, complete blood count (CBC), urinalysis, urine toxicology, blood chemistries, and tests for liver and kidney function, HIV-1, hepatitis B, and hepatitis C. Subjects were excluded if they had significant medical problems detected by history, physical examination, or screening laboratory tests; if an HIV-1, hepatitis B, or hepatitis C test was positive; or if they had traveled to countries where ETEC or cholera infection is endemic within two years prior to receipt of investigational agent.

### Study design

The study was conducted between September 2011 and April 2012. The subjects were admitted into the 30-bed CIR Isolation Unit. ETEC strain H10407 was used as the challenge strain, and all subjects received a challenge dose of either 10^5^ or 10^6^ CFU of this virulent organism. There were no placebo recipients in the study. A total of 30 subjects were challenged. The participants were divided randomly (1:1) into two groups of 15 subjects per group in a double-blinded manner (S1 Text). Group A received a dose of 1x10^5^ and group B received a dose of 1x10^6^ CFU. Both the groups received bicarbonate buffer (2 gms) with the dose. All subjects fasted overnight (~9 hours) before dosing and for 90 minutes after dosing.

### Challenge strain

The challenge strain H10407 is ETEC serotype O78:H11, produces heat labile toxin (LT) and two forms of heat stable toxin (STh and STp). The strain also produces colonization factor I (CFA/I). It is sensitive to ampicillin, trimethoprim-sulfamethoxazole, and ciprofloxacin, which are used typically to treat ETEC H10407 infections. A current good manufacturing practices (cGMP) quality production cell bank (PCB) for ETEC H10407 was prepared by the Walter Reed Army Institute of Research (WRAIR) Pilot Bioproduction Facility (Silver Spring, MD). The manufacturing information and production records for the PCB of these strains were provided to the FDA under BB-IND-7766 (batch production record 285–000, lot number. 0519). The ETEC H10407 challenge strain was stored in 2-ml cryostorage tubes (1 ml per tube) held at -80°C ±10°C in the bio-production facility at WRAIR. Cryovials containing organisms from this PCB were transferred on dry ice from WRAIR to the CIR Enterics Research Laboratory, JHBSPH, and stored at -80°C±10°C in a locked and temperature-monitored freezer.

### Inocula and challenge

The challenge inocula were prepared as described previously [17] with modifications to achieve the expected doses. The inocula were prepared from fresh, plate-grown organisms, using a study-specific procedure. The number of CFU in the inocula was validated by titrating and plating on Luria agar plates (Becton, Dickinson and Company, Sparks, MD) before and after administration to volunteers. A sample of the final inoculum was also examined by Gram stain for purity and by agglutination in anti-H10407 antiserum before being administered to subjects.

The bicarbonate buffer was prepared from USP-grade sodium bicarbonate (Fisher Scientific, Fair Lawn, NJ) by dissolving 13.35 g of sodium bicarbonate in 1,000 ml of sterile water for irrigation (Hospira, Inc., Lake Forest, IL). As with the earlier studies [17], the bacterial challenge was administered in bicarbonate buffer after an overnight (9 hours) fast. At approximately 0900 h on the day of challenge, subjects drank 120 ml of the sodium bicarbonate buffer to neutralize gastric acidity. Approximately 1 min later, subjects drank the ETEC H10407 inoculum dissolved in 30 ml of the same buffer solution.

### Clinical outcome evaluation

Medical interviews and physical examinations were performed daily and additional medical assessments and vital sign measurements were performed by the study team 3 times daily during the inpatient stay. Active solicitation regarding the following symptoms took place during the medical interview: fever, vomiting, nausea, abdominal pain, abdominal cramping, myalgia, malaise, bloating, flatulence, headache, lightheadedness, chills, constipation, and anorexia. Fever was defined as an oral temperature of 100.4°F or above. Severity of fever, vomiting, other symptoms and grades of stools were categorized as below [17]. Diarrhea was defined as 1 loose/liquid stool (grade 3) of 300 g in any 24-hour period or 2 loose/liquid stools totaling 200 g during any 48-hour period within 120 h of challenge with ETEC H10407. Diarrhea was classified as mild (1 to 3 diarrheal stools totaling 200 to 400 g/24 h), moderate (4 to 5 diarrheal stools or 401 to 800 g/24 h), or severe (6 or more diarrheal stools or 800 g/24 h). The subjects were treated with either oral rehydration solution (Ceralyte; Cera Products, Inc., Columbia, MD) or intravenous (i.v.) fluid or administration of antibiotics as per protocol guidelines. All the subjects were treated with ciprofloxacin (500 mg twice daily) approximately 120 h after challenge except those who meet the criteria for early antibiotic therapy and were treated earlier. To be eligible for discharge, subjects needed to have at least 2 negative stool cultures for ETEC H10407. Subjects were seen as outpatients in the clinic 10 and 28 days after challenge and contacted by telephone about 3 months after challenge.

### Fecal microbiology

Post challenge shedding levels of the challenge strain ETEC H10407 was detected in the fecal samples [17, 18]. Qualitative and quantitative microbiology assessment were done as described [17]. For qualitative assessment, up to 5 colonies appearing to be *E*. *coli* on MacConkey agar plates were tested for agglutination with antiserum to H10407. For semiquantitative microbiology, the fecal sample was serially diluted and spread onto MacConkey agar. After overnight incubation, the concentration of bacteria which appeared to be *E*. *coli* was calculated, and the proportion of these colonies (of 5 colonies tested) which agglutinated with anti-H10407 antisera was recorded. For analysis, subjects who did not shed H10407, a value of 1 was used in place of 0 to calculate the geometric mean (GM). If a subject had a positive qualitative sample, but negative quantitative sample, a value of 500 (corresponding to half of the lowest detectable limit of the quantitative assay) was used for the GM calculation.

### Blood and fecal immunology

Blood and fecal specimens were collected from the challenged subjects to measure systemic and mucosal immune responses. Venous blood from subjects was collected in BD Vacutainer cell preparation tubes (CPT) with heparin (Becton Dickinson, Franklin Lakes, NJ, USA) and processed for the antibody in lymphocyte supernatant (ALS) assay as described before [8]. Briefly, peripheral blood mononuclear cells (PBMCs) were isolated and resuspended at 1x10^7^ viable lymphocytes per ml. PBMCs were then incubated for 72 h with no antigenic stimulation. The supernatant fluid was cryopreserved and subsequently used in an enzyme linked immunosorbent assay (ELISA) to measure the concentration of antibody released by the PBMCs. The ALS samples were tested for antigen- specific IgA. An ALS response was defined as a ≥4-fold increase in antigen-specific IgA antibody titer over the baseline. Pre- and postchallenge venous blood samples were collected and processed for serum [8, 17]. A serum response was defined as a ≥2.5-fold increase over the baseline. Antibodies from fecal samples were extracted as described before [8, 19, 20]. Thawed stool sample was mixed with protease inhibitor cocktail. Next, the mixture was centrifuged at 12,000 x *g* for 30 min. Aliquots of the supernatant were stored at -80°C until they were assayed by ELISA for total and specific IgA antibody contents.

ELISAs with serum, ALS, or fecal samples were performed according to standard protocols [8, 19]. In short, 96 well microtiter plates were coated with purified CFA/I or LPS diluted in PBS. A GM1-ELISA method was used for the determination of levels of LT-specific antibodies [8]. The plates were blocked and washed. Serum, ALS, and fecal samples were diluted 3-fold in the plates using 0.1% BSA-PBS-Tween as a diluent. Anti-human IgG or anti-human IgA conjugated with horseradish peroxidase (KPL, Baltimore, MD) followed by *o*-phenylenediamine dihydrochloride (OPD) (Sigma, St. Louis, MO) was added to each well. After 20 min, the plates were read at 490 nm in an automated ELISA reader. Levels of specific and total IgA for fecal samples were determined [8, 19]. Antibody titers for fecal specimens were expressed as units per milligram of IgA, and these titers were calculated by using the specific titer (in units per milliliter) divided by the total IgA contents (in micrograms per milliliter) and then multiplying by 1,000. The total IgA contents for given samples were determined by ELISA using a standard IgA preparation (Sigma, St. Louis, MO) with a known IgA concentration (1 mg/ml). Titers were calculated to interpolate the dilution of serum, which yielded an optical density (OD) above baseline of 0.2 for serum samples and of 0.4 for ALS and fecal samples. Prechallenge and postchallenge sera were tested simultaneously in the same plate. GM1 and LTB antigens were purchased from Sigma (Sigma- Aldrich, St. Louis, MO), and CFA/I and LPS antigens were obtained from the laboratory of Ann Mari Svennerholm (University of Gothenburg, Gothenburg, Sweden).

### Statistical analyses

Chi-square and *t*-tests were used to determine differences between groups as appropriate for categorical and continuous variables. Results of statistical analyses were considered significant only if *p*-values were less than 0.05. We used GraphPad Prism (GraphPad, CA) software to analyze the results.

## Results

### Demographics

**Fig 1:**
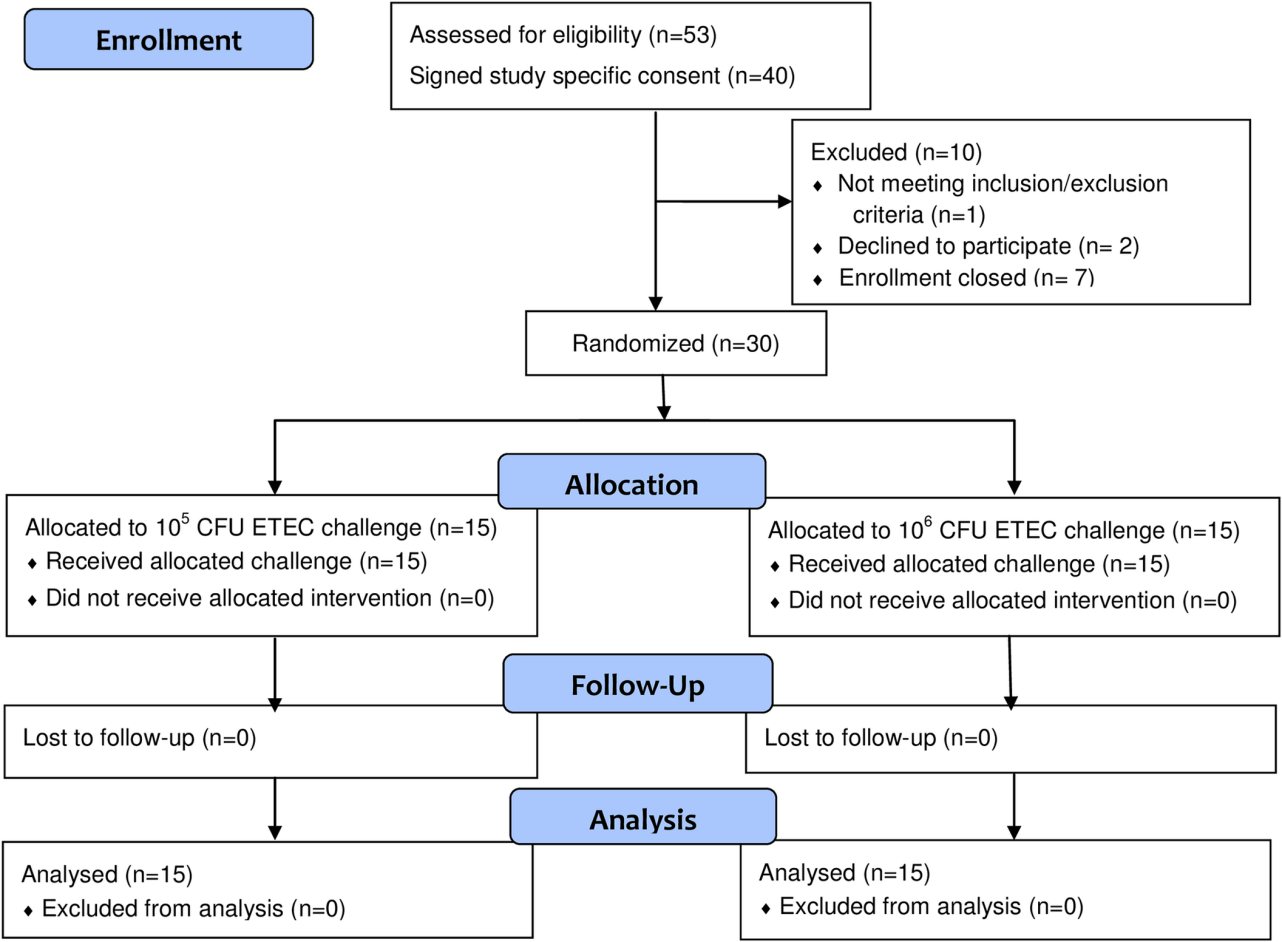
Flow chart of subject enrollment and retention in the study.

A total of 30 ETEC H10407-naïve subjects (based on possible exposure history solicited at screening) were enrolled in the study and randomly assigned to one of 2 dosing groups (Fig 1). Both groups had similar demographic characteristics (Table 1).

**Table 1:** Demographics of volunteers.

	Group A (n = 15) Dose = 1x10^5^	Group B (n = 15) Dose = 1x10^6^
**Mean Age (Range)**	**31.8 (24–42)**	**33.7 (23–45)**
**Females**	**4**	**3**
**Males**	**11**	**12**
**Black**	**14**	**12**
**White**	**1**	**2**
**Other**	**0**	**1**
**Mean BMI (Range)**	**26.5 (21.9–37.2)**	**32.3 (23.1–51.4)**

### Clinical symptoms

Clinical outcomes and selected solicited adverse events for both groups are summarized in Table 2 and S2 Table respectively. The number of subjects with MSD was somewhat higher in group B, 4/15 (26.7%) compared to 2/15 (13.3%) in group A. Four of these subjects with MSD were given early antibiotics (before day 5 after challenge). There was 1 subject with mild diarrhea in each group. The mean incubation period from challenge to initiation of diarrhea was similar in both the groups (~60hrs) (Table 2) and somewhat longer than reported previously for higher H10407 challenge doses (~48hrs) (10, 17). The mean total diarrhea stool output and mean maximum diarrhea stool output in 24 hours were 2.5 fold and 1.9 fold higher in group B than in group A, respectively (Table 2). The mean duration of diarrhea was slightly higher in group B, 53 hours compared to 48 hours in group A. The mean maximum number of diarrhea stools in 24 hours was 10 in group B and 4 in group A (Table 2). Following the challenge, there were no serious unexpected adverse events (S2 Table). The numbers of adverse events were slightly higher in group B than group A. Four of the 5 subjects with grade 2 or 3 vomiting were from group B. Six subjects from group B and two subjects from group A had moderate to severe nausea. One subject in each group had a grade 1 fever (S2 Table).

**Table 2:** Clinical outcomes of the subjects challenged with ETEC H10407.

Clinical Outcomes	Group A Dose = 1x10^5^ (n = 15)	Group B Dose = 1x10^6^ (n = 15)
**Number with diarrhea (%)**	**3 (20.0%)**	**5 (33.3%)**
**Number with moderate or severe diarrhea (%)**	**2 (13.3%)**	**4 (26.7%)**
**Mean incubation period in hours (range) Median**	**61 (32–79) 71**	**60 (38–85) 58**
**Mean diarrhea stool output in 24-hour period in grams (range) Median**	**667** **(328–1259)** **415**	**1268** **(269–1900)** **1245**
**Mean diarrhea total stool output in grams (range)** **Median**	**876** **(328–1599)** **702**	**1755** **(269–2423)** **2211**
**Mean duration of diarrhea in hours (range) Median**	**48 (0–87) 56**	**53 (43–69) 50**
**Mean of the maximum number of stools in 24 hours (range) Median**	**4 (1–9) 2**	**10 (3–29) 5**

### Shedding of the challenge strain

All but one of the 30 challenged subjects shed ETEC H10407 in their stool post challenge. The subject who did not shed was given the higher dose and this individual also did not have any gastrointestinal or systemic signs and symptoms associated with enteric illness. Five subjects did not start shedding the challenge organism until day 3 post challenge. Three of these subjects were in group B and two in group A. These 5 subjects also did not have any diarrhea throughout the study period. Overall the GM of H10407 ETEC shed on day 2 post challenge was the same (1.1x10^5^ CFU) in both the groups while the GM of the maximum shedding was ~2 fold higher in the group B (1.8 x 10^6^) compared to group A (8.3 x 10^5^) (S3 Table) To analyze shedding levels by clinical outcome post-challenge, subjects were divided into three groups: Group 1 included subjects with MSD (n = 6), Group 2, subjects with mild diarrhea (n = 2) and Group 3, subjects with no diarrhea (n = 22) (S3 Table). The GM of H10407 ETEC shed on second day after challenge was 1.9x10^7^ CFU in the subjects with MSD, 1.4x10^5^ CFU in the subjects with mild diarrhea and 2.7x10^4^ CFU in the subjects with no diarrhea (p = 0.0360, MSD vs mild + no diarrhea). The GM for maximum shedding was also significantly higher in the subjects with MSD (p<0.0001 MSD vs mild + no diarrhea) (S3 Table).

### Post challenge immune responses

The systemic and mucosal antibody responses to the primary antigens (CFA/I and LTB) and “O” antigen of H10407 (O78) were compared in the 15 subjects challenged with 10^5^ dose (group A) and in the 15 subjects challenged with 10^6^ dose (group B). These comparisons were based on the frequency and magnitude of serum, ALS and fecal IgA antibody responses to the 3 antigens mentioned above.

#### LPS O78

**Fig 2:**
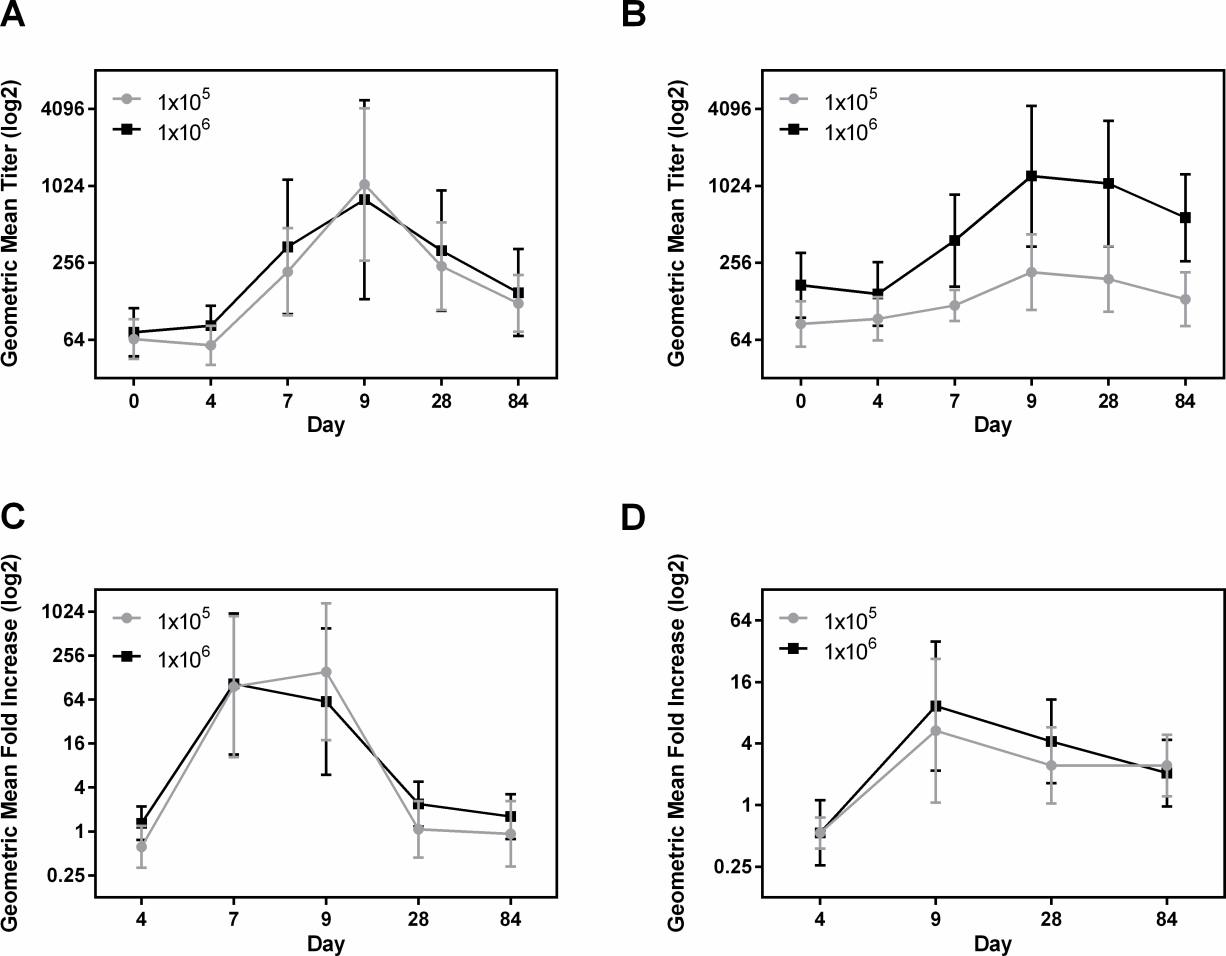
Dose dependent immune responses to LPS in serum, ALS and fecal extracts. [(A and B) geometric mean titers (95% confidence intervals) of antibody responses to LPS IgA in serum (A), IgG in serum (B) on the day before challenge (day 0) and on days 4, 7, 9, 28, and 84 following challenge with either 1x10^5^ or 1x10^6^ CFU doses of ETEC strain H10407. (C and D) geometric mean fold increase from day before challenge (95% confidence intervals), LPS IgA in ALS (C) on days 4, 7, 9, 28, and 84 and LPS IgA in fecal extracts (D) on days 4, 9, 28 and 84 following challenge. The titers are in log2 scale.].

The frequency of responders to IgA and IgG LPS in serum was similar in group A and B (Table 3). In both groups, the IgA and IgG responses to LPS in serum and IgA responses in fecal extracts peaked at day 9 post challenge (Fig 2). IgA-ALS responses peaked at day 7 in group B and at day 9 in group A (Fig 2). The magnitude of the responses to IgA in serum for the two groups was similar; whereas, the IgG GMT in group B subjects was significantly higher than among group A subjects on day 7 (p = 0.0128), day 9 (p = 0.0186), day 28 (p = 0.0086) and day 84 (p = 0.0014) (Fig 2A and 2B). As shown in Fig 2A, the GMT of LPS IgA in serum peaked on day 9 in both groups A and B (16.27 fold increase, p = 0.0091 in group A and 10.90 fold increase in group B compared to baseline). The GMTs for anti-LPS IgG responses were also significantly increased on day 9 over baseline, but the GMT for IgG was higher in group B (2.54 fold increase, p = 0.0292 in group A and 7.1 fold increase, p = 0.0051 in group B). (Fig 2B)

**Table 3:** Response rates^a^ against IgA and IgG of LPS O78 antigen by dose.

Antigens	Group A (1x10^5^, n = 15)		Group B (1x10^6^, n = 15)	
	IgA	IgG	IgA	IgG
**Serum**	**9 (60.0%)**	**9 (60.0%)**	**7 (46.7%)**	**9 (60.0%)**
**ALS**	**10 (66.7%)**		**12 (80.0%)**	
**Fecal**	**9 (60%)**		**11 (73.3%)**	

^a^For serum a 2.5 fold or greater and for ALS and fecal samples a 4 fold or greater rise in titers from baseline were considered a response.

IgA responses to LPS in ALS and fecal extracts were dose dependent (Table 3). The GMT of ALS responses in both the groups were very similar, increased 125.8 fold (p = 0.0012) on day 9 and 116.3 fold (p = 0.0003) on day 7 in group A and B respectively over baseline (Fig 2C). ALS responses declined to baseline levels by day 28 post challenge in both the groups. Anti-O78 specific IgA GMTs in fecal extracts were peaked at day 9 and increased 5.33 fold (p = 0.0367) and 9.32 fold (p = 0.0229) over baseline following challenge in groups A and B respectively (Fig 2D).

### CFA/I

**Fig 3:**
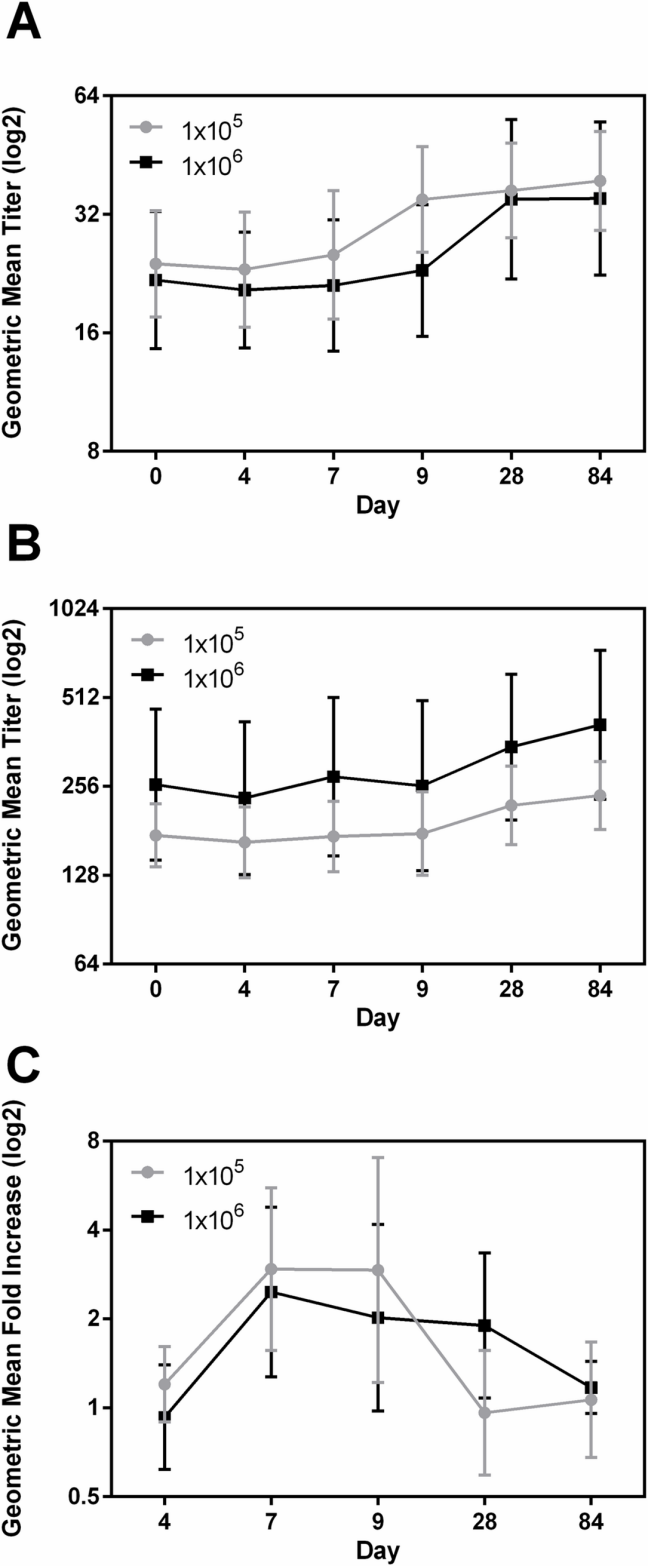
Dose dependent immune responses to CFA/I in serum and ALS. [(A and B) geometric mean titers (95% confidence intervals) of antibody responses to CFA/I IgA in serum (A), IgG in serum (B) on the day before challenge (day 0) and on days 4, 7, 9, 28, and 84 following challenge with either 1x10^5^ or 1x10^6^ CFU doses of ETEC strain H10407. (C) Geometric mean fold increase from day before challenge (95% confidence intervals) of CFA/I IgA in ALS on days 4, 7, 9, 28, and 84 following challenge. The titers are in log2 scale.].

The highest response frequency for CFA/I IgA was found in ALS. The response frequencies in serum IgA and IgG were low and dose dependent, however, in ALS the frequency of responses to IgA were higher in group A compared to group B. (Table 4). For fecal extracts 4 (26.7%) subjects in each group mounted anti-CFA/I IgA responses (Table 4). The magnitude of IgA and IgG responses in serum and IgA in ALS (Fig 3) and fecal samples across both dosing groups were not significantly different. Interestingly, unlike other antigens, the serum GMT either plateaued (IgA) or continued to increase at day 84 (IgG) (Fig 3A and 3B). The GMT of ALS IgA responses peaked at day 7 (fold increase 2.9, p = 0.0028) and remained high until day 9, and dropped to baseline by day 28 in group A. However, the GMT also peaked at day 7 (fold increase 2.5) in group B, but did not return to baseline levels until day 84 (Fig 3C). The GMT for fecal extracts did not increase above baseline levels in either dosing groups (S1A Fig).

**Table 4:** Response rates^a^ against IgA and IgG of CFA/I antigen by dose.

Antigens	Group A (1x10^5^, n = 15)		Group B (1x10^6^, n = 15)	
	IgA	IgG	IgA	IgG
**Serum**	**1 (6.7%)**	**2 (13.3%)**	**3 (20.0%)**	**4 (26.7%)**
**ALS**	**10 (66.7%)**		**7 (46.7%)**	
**Fecal**	**4 (26.7%)**		**4 (26.7%)**	

^a^For serum a 2.5 fold or greater and for ALS and fecal samples a 4 fold or greater rise in titers from baseline were considered a response.

### LTB

**Fig 4:**
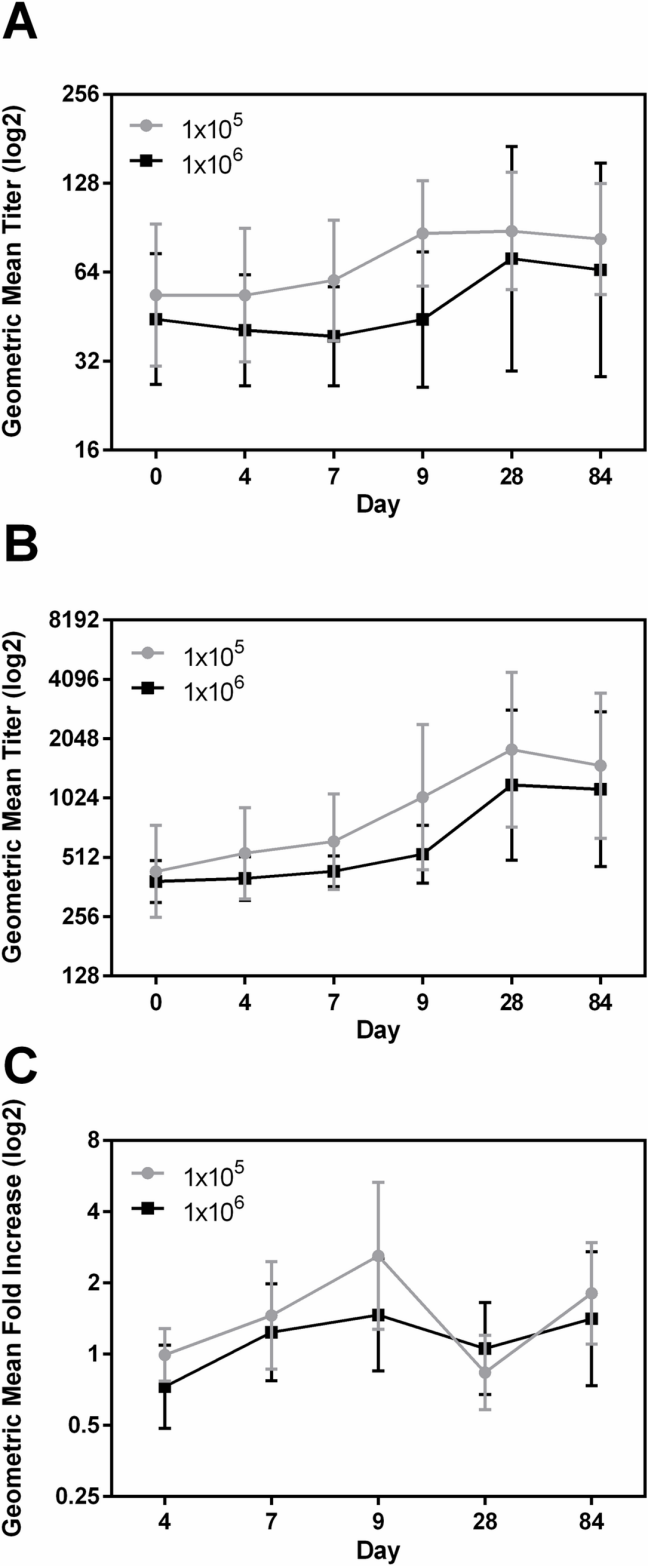
Dose dependent immune responses to LTB in serum and ALS. [(A and B) geometric mean titers (95% confidence intervals) of antibody responses to LTB IgA in serum (A), IgG in serum (B) on the day before challenge (day 0) and on days 4, 7, 9, 28, and 84 following challenge with either 1x10^5^ or 1x10^6^ CFU doses of ETEC strain H10407. (C) Geometric mean fold increase from day before challenge (95% confidence intervals) of LTB IgA in ALS on days 4, 7, 9, 28, and 84 following challenge. The titers are in log2 scale.].

The frequency and magnitudes of anti-LTB antibody responses were lower than LPS but higher than CFA/I in both the groups. For serum, frequency of responses to anti-LTB specific IgA and IgG were similar in the two doses (Table 5). However, in ALS, similar to CFA/I, the frequency of responses to anti LTB IgA was higher in group A compared to group B. (Table 5). The GMT of the magnitude of responses to IgA also tended to be higher in group A compared to group B in both serum and ALS, but they were not significantly different (Fig 4A, 4B and 4C). The serum IgA GMT of anti-LTB peaked at day 28 in group B (1.63 fold increase over baseline) and at day 9 in group A (1.60 fold increase over baseline) (Fig 4A and 4B). The ALS anti-LTB response peaked at day 9 in group A (2.60 fold increase from baseline titer, p = 0.021) and returned to baseline by day 28 but again increased at day 84 (2.29 fold increase from day 28 p = 0.015) (Fig 4C). Group B had similar kinetics with lower magnitude of response (maximum fold increase 1.44 over baseline) (Fig 4C). The titers in the fecal samples were very low and were not different between the two groups (S1B Fig).

**Table 5:** Response rates^a^ against IgA and IgG of LTB antigen by dose.

Antigens	Group A (1x10^5^, n = 15)		Group B (1x10^6^, n = 15)	
	IgA	IgG	IgA	IgG
**Serum**	**3 (20.0%)**	**7 (46.7%)**	**4 (26.7%)**	**6 (40.0%)**
**ALS**	**9 (60.0%)**		**5 (33.3%)**	
**Fecal**	**4 (26.7%)**		**4 (26.7%)**	

^a^For serum a 2.5 fold or greater and for ALS and fecal samples a 4 fold or greater rise in titers from baseline were considered a response.

In general, in previous studies after challenge with ETEC, peak ALS IgA responses were most frequently observed at days 7 to 9 post-challenge [8, 20, 21, 22]. In this study we looked at an earlier time point at day 4 for ALS samples to find any earlier responses. With the exception of very few subjects the earliest responses were on day 9 in these low dose groups (Figs 2C, 3C and 4C).

### Immune responses in the subjects with and without clinical symptoms

We compared immune responses in the subjects that had MSD, with subjects who had no diarrhea (ND) irrespective of the doses. There were 6 subjects with MSD, 22 with ND and 2 with mild diarrhea. Since there were only two subjects with mild diarrhea we have included those two subjects in the ND group for ease of analysis.

### LPS O78

**Fig 5:**
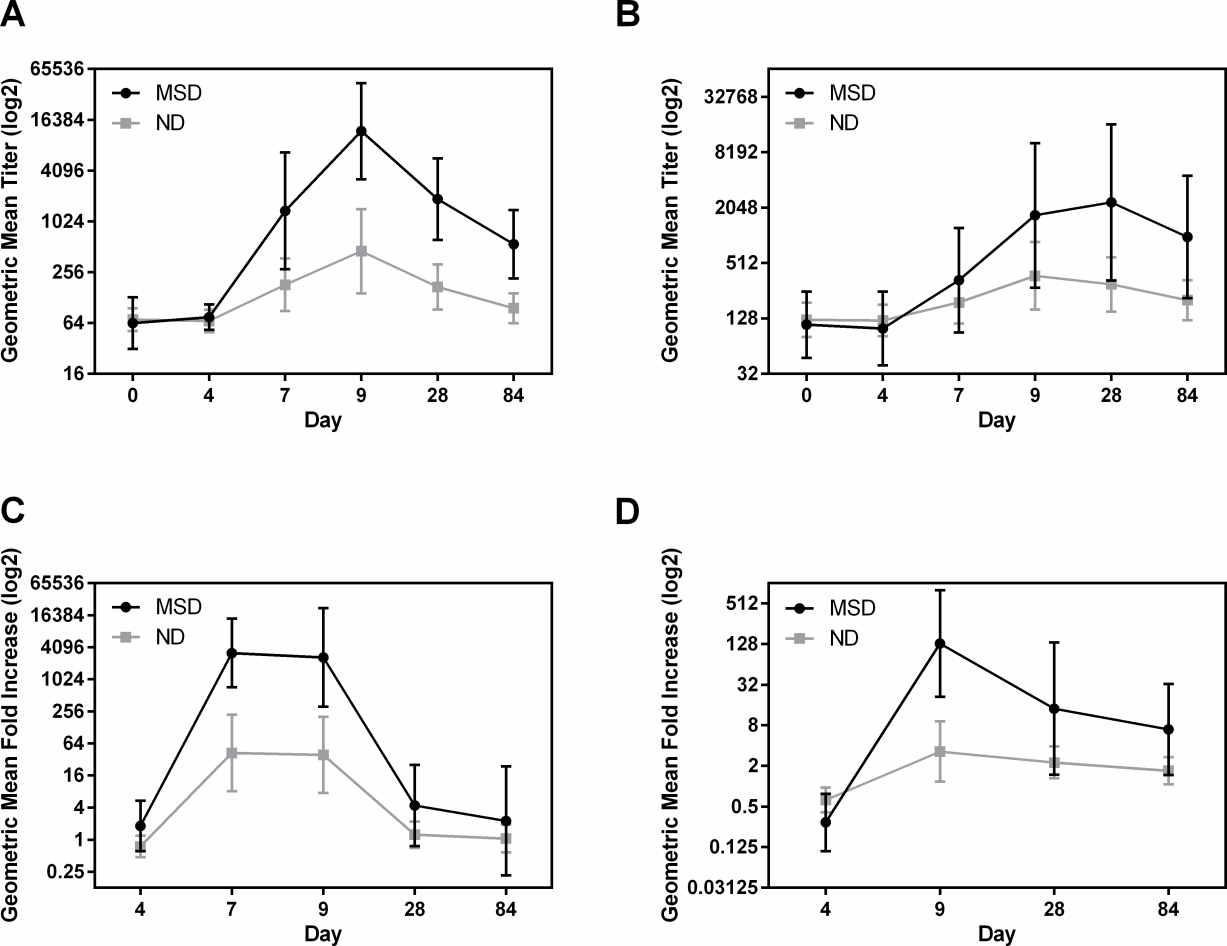
Diarrhea severity dependent immune responses to LPS in serum, ALS and fecal extracts. (A and B) geometric mean titers (95% confidence intervals) of antibody responses to LPS IgA in serum (A), IgG in serum (B) on the day before challenge (day 0) and on days 4, 7, 9, 28, and 84 following challenge in the MSD and ND groups. (C and D) geometric mean fold increase from day before challenge (95% confidence intervals), IgA in ALS (C) on days 4, 7, 9, 28, and 84 and IgA in fecal extracts (D) on days 4, 9, 28 and 84 following challenge. The titers are in log2 scale.].

All (100%) of the MSD group responded to LPS IgA and IgG in serum. By contrast, only 10 (41.7%) and 12 (50%) subjects in the ND group mounted anti- IgA and IgG responses to LPS in serum respectively (Table 6). For ALS and fecal IgA responses, again more subjects responded in the MSD group than among the ND group (Table 6). The GMT of the anti-O78 serum IgA response in the MSD group was significantly higher at day 9 (p = 0.044), 28 (p = 0.001) and 84 (p = 0.0006) compared to the ND group (Fig 5A). The GMT of IgG peaked on day 28 and increased 188.47 fold (p = 0.002) in the subjects with MSD while increased only 6.76 fold (p = 0.029) among the subjects with no or mild diarrhea (Fig 5B). The GMT of the anti-O78 in ALS peaked at day 7 in both the groups however increased 3184.30 fold over baseline in the MSD group; while it increased only 42.14 fold over baseline in the ND group (Fig 5C). In fecal extract the GMT of LPS IgA significantly increased to 10.20 fold over baseline at day 9 (p = 0.0022) in the MSD group; while there was no increase in GMT titer in the ND group (Fig 5D). The GMT of LPS IgA in fecal extracts was significantly higher in the MSD group compared to the ND group (at day 9, p = 0.0064).

**Table 6:** Diarrhea severity dependent response rates^a^ against IgA and IgG of LPS antigen.

Antigens	No Diarrhea (ND) n = 24		Moderate to severe diarrhea (MSD) n = 6	
	IgA	IgG	IgA	IgG
**Serum**	**10 (41.7%)**	**12 (50.0%)**	**6 (100.0%)**	**6 (100.0%)**
**ALS**	**16 (66.7%)**		**6 (100.0%)**	
**Fecal**	**14 (58.3%)**		**6 (100.0%)**	

^a^For serum a 2.5 fold or greater and for ALS and fecal samples a 4 fold or greater rise in titers from baseline were considered a response.

### CFA/I

**Fig 6:**
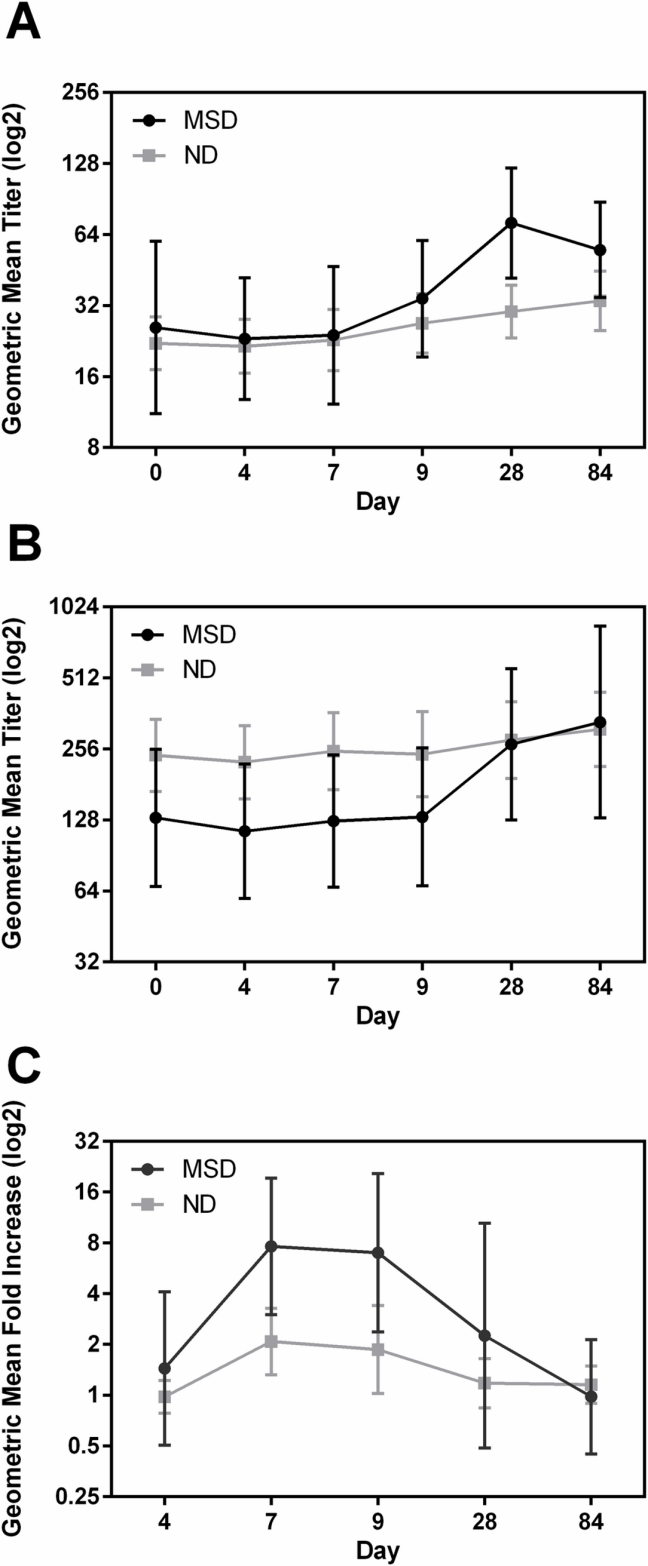
Diarrhea severity dependent immune responses to CFA/I in serum and ALS. [(A and B) geometric mean titers (95% confidence intervals) of antibody responses to CFA/I IgA in serum (A), IgG in serum (B) on the day before challenge (day 0) and on days 4, 7, 9, 28, and 84 following challenge in the MSD and ND groups. (C) Geometric mean fold increase from day before challenge (95% confidence intervals) of CFA/I IgA in ALS on days 4, 7, 9, 28, and 84 following challenge. The titers are in log2 scale.].

Responses to CFA/I in serum were lower than responses to LPS. Serum, ALS and fecal responses to CFA/I were more common in the MSD group. (Table 7). The GMT of the serum IgA response in the MSD group increased 2.77 fold (p<0.032) at day 28 from baseline and was significantly higher (p<0.0004) than the GMT in the ND group (Fig 6A). By contrast anti-CFA/I serum IgG GMTs were similar between the MSD and ND groups. Interestingly, the anti-CFA/I serum IgG response in subjects with MSD evolved more slowly over time, with the peak GMT being reached at day 84 post-challenge (Fig 6B). For IgA ALS, the GMT in the MSD group increased 7.6 fold at day 7 (p = 0.002) over baseline and, similar to LPS, stayed at the same level until day 9, then decreased to baseline by day 28. By contrast, for the ND group, the IgA ALS GMT only increased 2.1 fold (p = 0.010) at day 7 compared to baseline (Fig 6C). The GMT of anti-CFA/I IgA was also significantly higher (at day 7, p<0.0132) than that seen in the ND group (Fig 6C). The GMT in fecal extracts never increased above the baseline in either group (S2A Fig).

**Table 7:** Diarrhea severity dependent response rates^a^ against IgA and IgG of CFA/I antigen.

Antigens	No Diarrhea (ND) n = 24		Moderate to severe diarrhea (MSD) n = 6	
	IgA	IgG	IgA	IgG
**Serum**	**2 (8.3%)**	**3 (12.5%)**	**2 (33.3%)**	**3 (50.0%)**
**ALS**	**11 (45.8%)**		**5 (83.3%)**	
**Fecal**	**10 (41.7%)**		**5 (83.3%)**	

^a^For serum a 2.5 fold or greater and for ALS and fecal samples a 4 fold or greater rise in titers from baseline were considered a response.

### LTB

**Fig 7:**
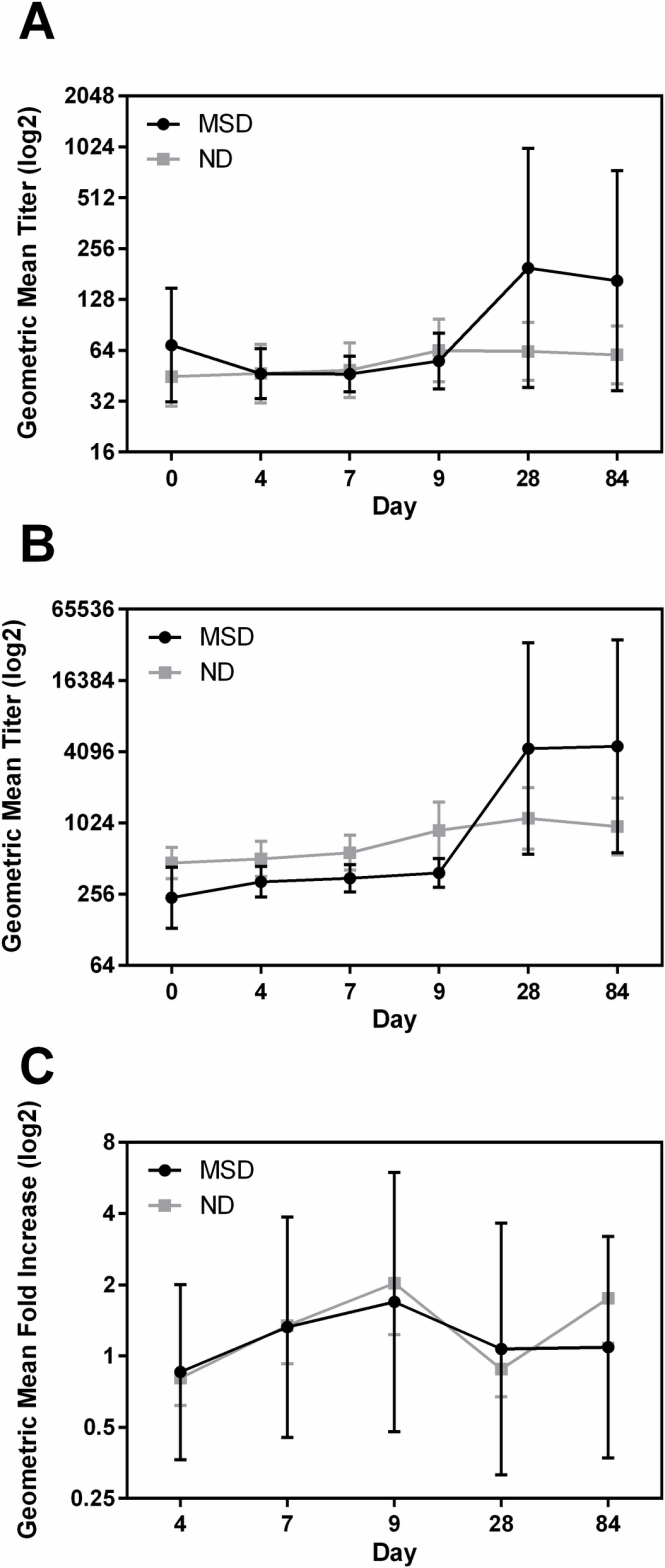
Diarrhea severity dependent immune responses to LTB in serum and ALS. [(A and B) geometric mean titers (95% confidence intervals) of antibody responses to LTB IgA in serum (A), IgG in serum (B) on the day before challenge (day 0) and on days 4, 7, 9, 28, and 84 following challenge in the MSD and ND groups. (C) Geometric mean fold increase from day before challenge (95% confidence intervals) of LTB IgA in ALS on days 4, 7, 9, 28, and 84 following challenge. The titers are in log2 scale.].

The responses to LTB IgA and IgG in serum and ALS followed the same trend as LPS and CFA/I, the MSD group having higher responses than the ND group (Table 8). By contrast, the response rates to LTB IgA in fecal extracts were higher in the ND group compared to MSD group (Table 8). Serum IgA GMT of LTB in the MSD group peaked at day 28 with 2.89 fold increase from baseline; the magnitude was higher than seen in the ND group. The serum IgG GMT peaked at day 28 in the MSD group, increased 18.98 fold (p = 0.0022) from baseline while the GMT only increased 2.39 fold in the ND group from baseline (Fig 7A and 7B). The magnitude of ALS IgA responses to LTB was low and similar in both the groups (Fig 7C). The GMT of IgA in ALS peaked at day 9, and increased only 1.69 fold in the MSD group and 2.02 fold (p = 0.033) in the ND group from baseline (Fig 7C). In the ND group the GMT of IgA responses in ALS was bimodal and increased again at day 84 (GMT of 2.0 fold increase from day 28 to 84, p = 0.038) (Fig 7C) similar to the dose dependent response (Fig 7C). The GMT in fecal samples never increased above the baseline in both the groups (S2B Fig).

**Table 8:** Severity dependent response rates^a^ against IgA and IgG of LTB antigen.

Antigens	No Diarrhea (ND) n = 24		Moderate to severe diarrhea (MSD) n = 6	
	IgA	IgG	IgA	IgG
**Serum**	**4 (16.7%)**	**7 (29.2%)**	**3 (50.0%)**	**6 (100.0%)**
**ALS**	**11 (45.8%)**		**3 (50.0%)**	
**Fecal**	**7 (29.2%)**		**1 (16.7%)**	

^a^For serum a 2.5 fold or greater and for ALS and fecal samples a 4 fold or greater rise in titers from baseline were considered a response.

### Baseline immune responses

We also analyzed the baseline GMTs (day before challenge) of all the antigens in serum, ALS and fecal extracts in the MSD and ND groups. The titers were similar in the two groups except the LTB IgG in serum was significantly higher in the ND group compared to the MSD group (p = 0.0211) (Table 9). The CFA/I IgG titer in serum was higher in the ND group but was not significantly different from the MSD group.

**Table 9:** Baseline titers in MSD and no diarrhea groups.

	Serum LPS IgA	Serum LPS IgG	ALSLPS IgA	Fecal LPS IgA	Serum LTB IgA	Serum LTB IgG* (p = 0.02)	ALS LTB IgA	Fecal LTB IgA	Serum CFA/I IgA	Serum CFA/I IgG	ALS CFA/I IgA	Fecal CFA/I IgA
**MSD**	**63.86**	**109.28**	**0.12**	**0.08**	**68.73**	**238.95**	**0.65**	**0.15**	**25.82**	**130.53**	**0.07**	**0.03**
**ND**	**70.14**	**123.48**	**0.12**	**0.14**	**44.78**	**468.47**	**0.75**	**0.17**	**22.16**	**240.24**	**0.09**	**0.04**

## Discussion

In this study, using the same buffering and fasting procedures applied in our prior challenge model refinement efforts [17] we found that the diarrhea attack rate was lower when H10407 was given to subjects at doses of 10^5^ (20%) or 10^6^ (33%) than the ~70% attack rate for MSD we observed in previous studies when this strain was given at a dose of 10^7^CFUs [17, 20]. We have used an attack rate for MSD of 50–70% as a reasonable barometer in our efforts to better optimize the ETEC human challenge model for use in evaluating preventive and therapeutic interventions for ETEC. Based on our observation in this most recent model refinement study, we conclude that a dose of 10^7^ H10407 remains the lowest practical dose for use in future volunteer studies evaluating the efficacy of candidate vaccines and other potential preventive tools targeting ETEC.

Nevertheless, this study did demonstrate that lower H10407 doses (10^5^ or 10^6^) were capable of causing MSD in some individuals. Although the mean incubation periods were similar for both the doses, the stool output and number of subjects with MSD were higher in 10^6^ dose. There were no unexpected serious adverse events in either group through the 180 day follow-up period. Solicited gastrointestinal and systemic signs and symptoms were only marginally higher in the 10^6^ group overall. Five subjects had vomiting and two had a mild fever.

In the study by Evans *et al* in 1978, [23] subjects were challenged with H10407, at a dose of 10^6^ after fasting for 5 hours, and there was no discernible clinical diarrhea reported in these subjects following dosing. In the present study we found some severe cases at this dose as well as at the lower 10^5^ dose suggesting that the overnight fast used in this study may have facilitated ETEC colonization and the subsequent development of illness. However, to determine the specific effect of the length of fasting period on clinical illness, a direct comparison of longer and shorter fasting periods is necessary. In our recent study, we have noted that an increased fasting time from 90 minutes before challenge to overnight can increase the diarrhea attack rate for another ETEC challenge strain, B7A (unpublished).

All the subjects except one shed the challenge strain even in this low dose challenge study. Although the overall shedding levels on day 2 were the same in both groups, the GM of the maximum shedding was higher in the subjects who received the higher dose. In this low dose study we found that 1.9x10^7^ CFU/gm of stool on day 2 after challenge corresponds to the development of MSD. A similar correlation between a threshold level of shedding and a higher risk for MSD was shown by us using both quantitative stool culture and q-PCR in a previous study [18]. Similarly, in our previous ETEC vaccine immunization and challenge study using a higher challenge dose of 10^7^ CFU of H10407, we also found 1.5x10^7^ CFU corresponds to MSD [20]. The association between level of colonization and diarrhea risk is also supported by several recent field studies in which quantitative TaqMan PCR has shown a similar threshold effect [4].

Not surprisingly, subjects challenged with H10407 seroconverted and had mucosal responses to known ETEC virulence antigens LPS, LTB and CFA/I. However, the frequency and magnitude of responses tended to be higher in subjects challenged with the 10^6^ dose compared to the 10^5^ dose suggesting a dose-dependent trend. The differences in response to these two doses varied depending on the antigen and sample types. Interestingly, responses to LTB were higher in the lower dose group compared to the higher dose group in all types of samples. In this low dose study the highest IgA and IgG responses to LPS in serum, ALS and fecal samples were generally at day 9. For LTB and CFA/I IgA and IgG, the peak responses were either on day 9 or day 28. Similar to our previous findings [8] with higher H10407 doses, the frequency and magnitude of responses to CFA/I were lower compared to LPS and LTB in all the samples. It is interesting to note that the responses to CFA/I IgG in serum peaked on day 7 and kept on increasing until day 84. The magnitudes of the IgA responses to LTB and CFA/I in fecal samples were negligible in both dose groups. In general, immune responses induced by the lower doses used in this study were more modest than those seen earlier when a higher dose of H10407 (~10^7^ CFU) was used. A more detailed comparison of the humoral and cellular immune responses induced across the full spectrum of H10407 challenges doses studies by our investigative team in recent years (10^8^ to 10^5^ CFU of H10407) is currently underway.

There is a growing concern of asymptomatic colonization of enteropathogens in the gut of the children in the endemic countries because of its impact on longitudinal public health issues, such as growth faltering, oral vaccine low efficacy and poor neurocognitive development through environmental enteropathy/environmental enteric dysfunction [24]. Asymptomatic ETEC infections are common in endemic countries and may impact the intestinal health as well as long-term developmental potential in the children in these areas [4, 25]. This low dose CHIM model of ETEC may serve as a tool to decipher these critical interactions. Furthermore, the levels of immunity induced by these asymptomatic ETEC infections is not well studied and needs to be better understood since these may contribute to protection in the field and may serve to diminish vaccine take. In this study we compared antigen-specific ETEC immune response profiles in the subjects with clinical and subclinical infections after challenge with low doses of ETEC which may better mimic more natural levels of exposure in the ETEC endemic areas. Notably, although of lower magnitude, there were considerable number of responders and increased titers in the subjects with no diarrhea. In this study we found that the magnitude of serum and mucosal antibody responses after challenge were symptom dependent with subjects developing MSD generally showing stronger responses than those experiencing asymptomatic colonization irrespective of the ETEC challenge dose.

Of note, the challenge bacteria were shed by all volunteers (except one) but the shedding level was related to the dose of the challenge and the severity of the symptoms. Thus, the magnitude of immune responses depended on the colonization levels, with the one exception being the responses to LTB where the responses were similar between those with MSD and those with ND.

In natural infection it is often assumed that some degree of pre-existing immunity to a given pathogen may contribute to an individual’s susceptibility or resistance to infection and subsequent illness. In the present study we compared baseline serum and mucosal antigen-specific antibody levels to see if they are predictive of challenge outcome. Of the antigens evaluated, only LTB IgG levels in serum were significantly higher in subjects with no post-challenge diarrhea compared to subjects with MSD. A similar relationship has been previously noted by Porter and colleagues in their recent systematic review of ETEC CHIMs studies [10]. However, further investigation is required to establish if LTB IgG levels in serum can serve as an indication of immunity to ETEC.

It is noteworthy that in our study, subjects with no history of exposure to ETEC and/or cholera (>2 years) given lower ETEC doses responded with very different clinical outcomes. Among those few subjects developing MSD, the incubation time was longer than has been generally seen at higher doses (~60 hrs versus 48 hrs) [10, 17] but the threshold level of shedding seen in these subjects was comparable to that previously associated with diarrheal illness in H10407 studies (≥10^7^CFU per gram of stool) [18, 20]; while subjects with mild or no illness exhibited much lower levels of ETEC shedding (approximately 10^4^ to 10^5^ CFU per gram of stool). The cellular and molecular basis for these differences deserve further investigation using new genomic, transcriptomic, proteomic, systems biology and microbiome tools. These studies are now underway [26, 27] in the hope that they will lead to better defining host factors (immunological or microbiological) that may contribute to ETEC susceptibility and immunity.

## Supporting information

S1 Text Supporting information on screening, randomization and blinding. (DOCX)

S1 Table CONSORT checklist. (DOC)

S2 Table Selected solicited adverse events in the two groups. (DOCX)

S3 Table Geometric Mean Titer (GMT) of H10407 shed on day 2 and maximum shedding. (DOCX)

S1 Fig Dose dependent immune responses to CFA/I and LTB IgA in fecal extracts. Geometric mean fold increases (95% confidence intervals) of antibody responses to CFA/I IgA in fecal (A), LTB IgA in fecal (B) from the day before challenge (day 0) and on days 4, 9, 28, and 84 following challenge with either 1x10^5^ or 1x10^6^ CFU doses of ETEC strain H10407. The titers are in log2 scale.
(TIF)

S2 Fig Diarrhea severity dependent immune responses to CFA/I and LTB IgA in fecal extracts. Geometric mean fold increases (95% confidence intervals) of antibody responses to CFA/I IgA in fecal (A), LTB IgA in fecal (B) from the day before challenge (day 0) and on days 4, 9, 28, and 84 following challenge with either 1x10^5^ or 1x10^6^ CFU doses of ETEC strain H10407. The titers are in log2 scale.
(TIF)
