# Supplementary material for: Impact of lower challenge doses of enterotoxigenic Escherichia coli on clinical outcome, intestinal colonization and immune responses in adult volunteers
Source: PLoS Negl Trop Dis. 2018 Apr 27;12(4):e0006442. doi: 10.1371/journal.pntd.0006442 (PMC5942845; doi:10.1371/journal.pntd.0006442)
Supplement: S1 Text — (DOCX) [file pntd.0006442.s001.docx]

**Screening, randomization and blinding procedures**

Screening

On the screening days (study day -60 to -2) potential subjects were given a complete description of the study. To ensure comprehension of the study all subjects had to pass a written examination before inclusion in the study (minimum passing grade is 70%). All missed questions were reviewed with subjects to further emphasize important points. Following successful completion of the comprehension exam, a medical history was obtained along with a series of clinical laboratory examinations to rule out occult illness and pregnancy. Hematology, serum chemistry, hepatitis B antigen, hepatitis C antibody, HIV-1 antibody, serum IgA level, serum HCG pregnancy test (for females only), urinalysis and urine toxicology (drug screening) will be used for screening purposes.

Subjects who met all inclusion criteria and none of the exclusion criteria, pass the comprehension test, and sign the study consent form were eligible for the study.

Randomization, group assignment and masking

Subjects were randomized to one of two different dose groups. Enrollment was performed online using the enrollment module of AdvantageEDC^SM^, The EMMES Corporation’s electronic data capture system. The coded treatment assignments were included in the enrollment module for the trial. Each subject enrolled in the trial was assigned to a coded treatment assignment after demographic and eligibility data have been entered into the system. The unblinded pharmacist was provided with the treatment assignment codes for preparation of the challenge preparation to be given to each subject. The group assignment was randomized based on their subject ID number. The research pharmacist prepared the doses in a manner which the clinical staff cannot witness. Thus, the dose level was blinded to the clinical staff assessing clinical outcomes. The research pharmacist maintained the treatment code list in a secure place.

Subjects received the challenge inoculum in the drinking cup bearing their assigned subject identification number. Study identification numbers identified all samples for laboratory analyses.

Sample size calculation

The expected attack rates for 15 subjects in each dose arm and corresponding confidence intervals (CIs) are shown in the Table below. Since the lower bound of the 95% CI for the dose of 10^7^ was 53%, an attack rate of 33% (5 illnesses out of 15 volunteers) with either of the two doses under testing (10^6^ and 10^5^) will be significantly lower than that observed with the 10^7^ dose. While in previous studies a dose response on the attack rate has not yet been ascertained, the lowest dose (10^7^) tested resulted in a significantly lower rate of severe diarrhea as compared with the higher doses of 10^8^ and 10^9^. In this study we expect to observe a lower attack rate combined with diminished diarrhea severity in both groups, and thus be able to identify a challenge inoculum to use in subsequent vaccination-challenge studies.

| **15 Subjects per Cohort** | | |
| --- | --- | --- |
| **No. of ill subjects** | **Attack Rate** | **Exact two-sided 95% CI** |
| 2 | 0.13 | [0.02, 0.27] |
| 4 | 0.27 | [0.08, 0.39] |
| 5 | 0.33 | [0.15, 0.49] |
| 6 | 0.40 | [0.17, 0.528] |
| 8 | 0.53 | [0.23, 0.59] |
| 10 | 0.67 | [0.31, 0.69] |
| 12 | 0.80 | [0.41, 0.77] |
| 14 | 0.93 | [0.51, 0.85] |
