# Supplementary material for: Impact of lower challenge doses of enterotoxigenic Escherichia coli on clinical outcome, intestinal colonization and immune responses in adult volunteers
Source: PLoS Negl Trop Dis. 2018 Apr 27;12(4):e0006442. doi: 10.1371/journal.pntd.0006442 (PMC5942845; doi:10.1371/journal.pntd.0006442)
Supplement: S2 Table — (DOCX) [file pntd.0006442.s003.docx]

**S2 Table. Selected solicited adverse events in the two groups**

|  | **Group A (10^5^ dose)**  **N = 15** | | **Group B (10^6^ dose)**  **N = 15** | |
| --- | --- | --- | --- | --- |
| **Adverse Event** | **Total Number** | **Number with Moderate or Severe AE** | **Total Number** | **Number with Moderate or Severe AE** |
| **Loose Stool** | 4 | 0 | 4 | 0 |
| **Diarrhea** | 3 | 2 | 5 | 4 |
| **Nausea** | 4 | 2 | 8 | 6 |
| **Vomiting** | 2 | 1 | 4 | 4 |
| **Myalgia** | 0 | 0 | 0 | 0 |
| **Fever** | 1 | 0 | 1 | 0 |
| **Abdominal Pain** | 4 | 2 | 4 | 3 |
| **Abdominal Cramps** | 4 | 3 | 6 | 3 |
| **Malaise** | 5 | 1 | 3 | 3 |
| **Bloating** | 3 | 1 | 8 | 2 |
| **Flatulence** | 7 | 0 | 8 | 2 |
| **Headache** | 6 | 3 | 5 | 1 |
| **Lightheadedness** | 2 | 0 | 2 | 0 |
| **Constipation** | 1 | 0 | 3 | 3 |
| **Chills** | 1 | 1 | 0 | 0 |
| **Anorexia** | 4 | 2 | 5 | 3 |
| **Hypovolemia** | 0 | 0 | 0 | 0 |
| **Measured Temperature^1^** | 1 | 0 | 2 | 0 |
| **Results are based on the maximum severity per participant during the in-patient period (day 0 to 8).** | | | | |
