# Supplementary material for: Impact of lower challenge doses of enterotoxigenic Escherichia coli on clinical outcome, intestinal colonization and immune responses in adult volunteers
Source: PLoS Negl Trop Dis. 2018 Apr 27;12(4):e0006442. doi: 10.1371/journal.pntd.0006442 (PMC5942845; doi:10.1371/journal.pntd.0006442)
Supplement: S3 Table — (DOCX) [file pntd.0006442.s004.docx]

**S3 Table. Geometric Mean Titer (GMT) of H10407 shed on day 2 and maximum shedding**

|  | **Group A**  **Dose =1x10^5^** | **Group B**  **Dose =1x10^6^** | **No Diarrhea**  **n=22** | **Mild Diarrhea**  **n=2** | **Moderate / Severe Diarrhea**  **n=6** |
| --- | --- | --- | --- | --- | --- |
| **GMT day 2** | **1.1 x 10^5^** | **1.1 x 10^5^** | **2.7 x 10^4^** | **1.4 x 10^5^** | **1.9 x 10^7^** |
| **GMT Max** | **8.3 x 10^5^** | **1.8 x 10^6^** | **2.3 x 10^5^** | **2.7 x 10^6^** | **4.0 x 10^8^** |
| **No. with positive cultures** | **13** | **11** | **16** | **2** | **6** |
