# Supplementary figures and images for: Impact of lower challenge doses of enterotoxigenic Escherichia coli on clinical outcome, intestinal colonization and immune responses in adult volunteers
Source: PLoS Negl Trop Dis. 2018 Apr 27;12(4):e0006442. doi: 10.1371/journal.pntd.0006442 (PMC5942845; doi:10.1371/journal.pntd.0006442)

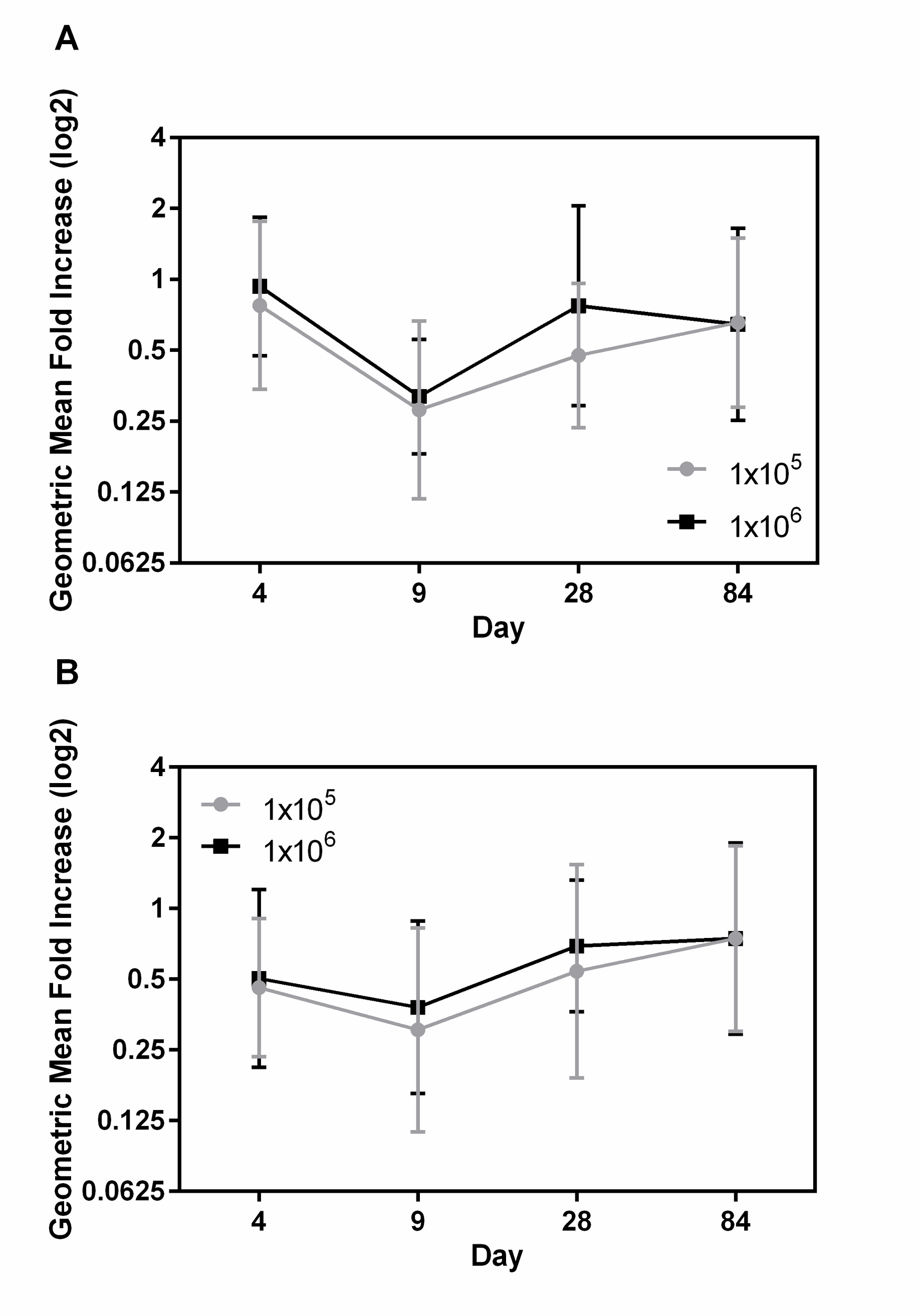

Supplement: S1 Fig — Geometric mean fold increases (95% confidence intervals) of antibody responses to CFA/I IgA in fecal (A), LTB IgA in fecal (B) from the day before challenge (day 0) and on days 4, 9, 28, and 84 following challenge with either 1x10^5^ or 1x10^6^ CFU doses of ETEC strain H10407. The titers are in log2 scale. (TIF) [file pntd.0006442.s005.tif]

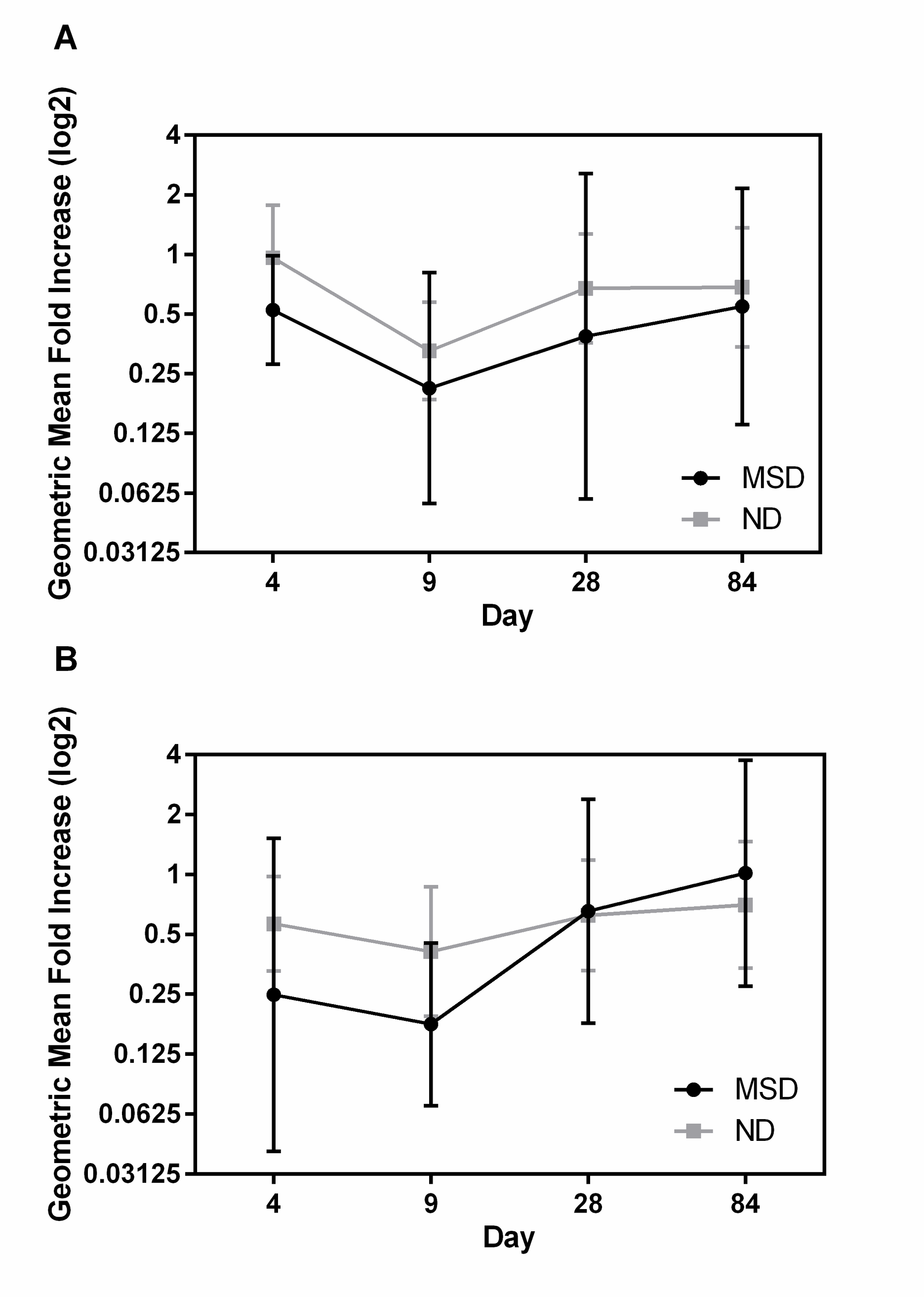

Supplement: S2 Fig — Geometric mean fold increases (95% confidence intervals) of antibody responses to CFA/I IgA in fecal (A), LTB IgA in fecal (B) from the day before challenge (day 0) and on days 4, 9, 28, and 84 following challenge with either 1x10^5^ or 1x10^6^ CFU doses of ETEC strain H10407. The titers are in log2 scale. (TIF) [file pntd.0006442.s006.tif]
